# The Effects of Volunteering on Hypertension by Health Insurance Status Among U.S. Adults Aged 50-64

**DOI:** 10.1093/geroni/igaf122.205

**Published:** 2025-12-31

**Authors:** Byeongju Ryu, Seoyoun Kim, Halvorsen Cal

**Affiliations:** Washington University in St. Louis, St. Louis, Missouri, United States; University of Michigan, Ann Arbor, Michigan, United States; Washington University in St. Louis, St. Louis, Missouri, United States

## Abstract

Hypertension is a major risk factor for cardiovascular disease, the leading cause of death in the U.S. Managing blood pressure can be challenging for individuals without health insurance due to barriers to healthcare access. Emerging evidence suggests that volunteering may serve as a public health intervention that reduces hypertension. However, it remains unclear whether the relationship between volunteering and hypertension differs by health insurance status. We analyzed data from the 2012–2018 Health and Retirement Study, restricting participants to those aged 50-64 (below Medicare eligibility age; N = 4,366). To address selection bias and ensure correct temporal order, we created entropy-balance weights using participants’ characteristics from 2012/2014 to balance volunteers and non-volunteers in 2014/2016. We then employed weighted Poisson regression with robust error variance to estimate the population average treatment effects of volunteering in 2014/2016 on hypertension in 2016/2018. We pooled waves because the outcome variable was measured in half the sample in consecutive waves. We ran seemingly unrelated estimations to statistically test for differences in effect sizes between insured and uninsured volunteers. Results indicate that volunteers were less likely to be hypertensive than non-volunteers in the full sample (IRR=0.91, p = 0.008). In subgroup analyses, volunteering was associated with a lower incidence of hypertension among both uninsured (IRR=0.70, p = 0.035) and insured (IRR=0.93, p = 0.035) respondents. These effects did not significantly differ (p = 0.104), suggesting that volunteers were less likely to be hypertensive regardless of insurance status. Our findings suggest that despite limited healthcare access, volunteering may be one pathway toward reducing hypertension in uninsured older adults.

